# British Colonial and Continental Homœopathic Medical Directory, 1898

**Published:** 1898-11

**Authors:** 


British, Colonial, and Continental Homœopathic Medical Directory, 1898. Edited by a member of the British Homœopathic Society and Dr. Alexander Villers, corresponding member of the British Homœopathic Society, London. Homœopathic Publishing Company, 12 Warwick Lane, Paternoster Row, E. C. Price, 2 shillings, or 50 cents.

This is the fourth yearly issue of this useful little book. It is intended, as the name shows, to be a guide in selecting homœopathic physicians in the different countries of Europe. It has been most carefully edited, the continental part being under the charge of that distinguished homœopathist, Dr. Alexander Villers. Physicians whose patients are traveling in Europe, and who wish to direct them to the care of homœopathists during their stay abroad, are recommended to possess themselves of a copy of this book.

